# Maternal Immune Activation Alters Fetal Brain Development and Enhances Proliferation of Neural Precursor Cells in Rats

**DOI:** 10.3389/fimmu.2020.01145

**Published:** 2020-06-09

**Authors:** Kelly J. Baines, Dendra M. Hillier, Faraj L. Haddad, Nagalingam Rajakumar, Susanne Schmid, Stephen J. Renaud

**Affiliations:** ^1^Department of Anatomy and Cell Biology, Schulich School of Medicine and Dentistry, University of Western Ontario, London, ON, Canada; ^2^Department of Psychiatry, University of Western Ontario, London, ON, Canada; ^3^Children's Health Research Institute, Lawson Health Research Institute, London, ON, Canada

**Keywords:** maternal immune activation, fetal brain development, PolyI:C, neural precursor cells, pregnancy

## Abstract

Maternal immune activation (MIA) caused by exposure to pathogens or inflammation during critical periods of neurodevelopment is a major risk factor for behavioral deficits and psychiatric illness in offspring. A spectrum of behavioral abnormalities can be recapitulated in rodents by inducing MIA using the viral mimetic, PolyI:C. Many studies have focused on long-term changes in brain structure and behavioral outcomes in offspring following maternal PolyI:C exposure, but acute changes in prenatal development are not well-characterized. Using RNA-Sequencing, we profiled acute transcriptomic changes in rat conceptuses (decidua along with nascent embryo and placenta) after maternal PolyI:C exposure during early gestation, which enabled us to capture gene expression changes provoked by MIA inclusive to the embryonic milieu. We identified a robust increase in expression of genes related to antiviral inflammation following maternal PolyI:C exposure, and a corresponding decrease in transcripts associated with nervous system development. At mid-gestation, regions of the developing cortex were thicker in fetuses prenatally challenged with PolyI:C, with females displaying a thicker ventricular zone and males a thicker cortical mantle. Along these lines, neural precursor cells (NPCs) isolated from fetal brains prenatally challenged with PolyI:C exhibited a higher rate of self-renewal. Expression of Notch1 and the Notch ligand, delta-like ligand 1, which are both highly implicated in maintenance of NPCs and nervous system development, was increased following PolyI:C exposure. These results suggest that MIA elicits rapid gene expression changes within the conceptus, including repression of neurodevelopmental pathways, resulting in profound alterations in fetal brain development.

## Introduction

Development of the brain is an intricately orchestrated process that commences early in gestation and is particularly vulnerable to changes in the prenatal environment. There is now substantial evidence that maternal infection during windows of susceptibility can affect critical aspects of fetal brain development, leading to a wide range of neuronal dysfunctions and behavioral outcomes in offspring (1). For example, epidemiological evidence implicates maternal infection as a major risk factor for neurodevelopmental disorders in offspring, including schizophrenia (2), autism-spectrum disorder (ASD) (3), and epilepsy (4), as well as neurodegenerative disorders including Alzheimer's disease and Parkinson's disease (5, 6). Altered brain development is recapitulated in offspring born to pregnant mice and rats exposed to different types of infections, resulting in a spectrum of behavioral abnormalities apparent in adolescent and adult offspring (7). Despite considerable progress identifying behavioral phenotypes in juvenile and adult offspring exposed prenatally to infection, the impact of infection on neuronal development and brain structure, particularly during fetal life, is not well-understood.

Maternal immune activation (MIA) in the absence of a pathogen is sufficient to elicit brain maldevelopment and behavioral abnormalities in offspring, indicating that the maternal response to infection, rather than the infection itself, is a likely culprit. One of the most recognized and established models of neurodevelopmental deficiencies resulting from acute exposure to MIA involves prenatal exposure to polyinosinic:polycytidylic acid (PolyI:C) (8). PolyI:C is a synthetic analog of double-stranded RNA, which resembles the molecular pattern of certain viruses. PolyI:C induces the generation of a cellular antiviral response, and stimulates the production of inflammatory cytokines including type I interferons, interleukin (IL)-6, and IL-1β, all of which may participate in MIA-induced neurodevelopmental impairment (9, 10). PolyI:C therefore effectively mimics the acute cellular response to viral infections and is advantageous as a model because the response is transient, and the stimulus intensity and timing can be precisely controlled to evaluate the impact of MIA during particular phases of brain development. Administration of PolyI:C to pregnant mice or rats during early-to-mid gestation or late gestation has revealed differing levels of sensitivity to fetal cytokines, corresponding to unique behavioral phenotypes later in life (11). These altered behaviors include deficits in social interaction, attention, memory, and sensorimotor function that have construct and face validity toward human neuropsychiatric disorders such as schizophrenia and ASD (12), suggesting that MIA may interfere with structural and developmental programming during prenatal brain development leading to behavioral changes in postnatal life.

The cerebral cortex integrates sensory and motor information and is responsible for shaping behavior, perception, higher-order thought, and reasoning in mammals. It develops from a single layer of neuroepithelium that lines the ventricles. As early as embryonic day 9 (E9) in rats, neural stem cells (NSCs) within the neuroepithelium undergo symmetric divisions to provide the early NSC pool that is crucial for determining cell number and adult brain size (13). By E11, NSCs start dividing asymmetrically to form neural precursor cells (NPCs), which then produce neurons that migrate away from the ventricular zone to form the preplate, and subsequent volley of neurons form the remainder of cortical layers. Recent evidence indicates that cortical neurogenesis is orchestrated by highly complex signaling mechanisms involving precise, temporally-regulated expression of transcription factors as well as the action of signaling molecules belonging to the Notch and Wnt pathways (14). Disruptions in maintenance of the NSC pool and cortical neurogenesis impact neuronal number and brain size, and also result in malformations in layering (heterotopia) and neuronal connections. It is also important to note that in rats, cortical angiogenesis begins at E10 and the blood-brain barrier is functional by E16. Consequently, early developmental periods prior to neuronal migration on E11 is a potentially vulnerable period for MIA-mediated abnormalities (15).

In this study, we injected pregnant rats with saline or PolyI:C at E8.5 and generated a transcriptional profile on whole conceptus tissue that revealed both the response of the nascent uterine environment to MIA and changes in gene expression critical for neurodevelopmental processes. We then evaluated cortical structure on E15.5 and isolated neurospheres to assess the self-renewal capacity of NSCs and NPCs. We discovered a robust antiviral inflammatory response following PolyI:C administration that correlated with impaired progression of neurodevelopmental events. Surprisingly, we also identified a “rebound effect” at mid-to-late gestation, in which cortical thickness was increased suggesting an expanded NSC pool that correlated to increased NSC/NPC proliferation capacity and altered expression of receptors and ligands regulating Notch signaling—a pathway crucial for NSC proliferation and nervous system development.

## Materials and Methods

### Animals

Female (6–8 weeks old) and male Sprague Dawley rats were obtained from Charles River Laboratories and maintained in a 12:12 h light-dark cycle (lights on at 7:00 a.m.) at constant temperature/humidity and food and water available *ad libitum*. Females were cycled by daily inspection of cells within the vaginal lavage and mated when in proestrus with a fertile male. E0.5 was defined as the day following mating if spermatozoa were detected within the vaginal lavage. All experiments were in compliance with guidelines outlined by the Canadian Council of Animal Care, using protocols approved by the University of Western Ontario Animal Care Committee.

### Injection Protocol and Tissue Collection

Pregnant females were injected intraperitoneally on E8.5 with sterile saline (0.9% NaCl) or an equal volume of saline containing 10 mg/kg PolyI:C sodium salt (Sigma-Aldrich). The 10 mg/kg dose of PolyI:C is based on our previous study (16), and other reports in which this dose of PolyI:C elicited an inflammatory response and altered behavioral phenotypes in offspring (17, 18). For experiments evaluating the acute impact of PolyI:C on gene expression in the conceptus (encompassing maternal decidua as well as nascent embryo and placenta), dams were euthanized 6 h following saline or PolyI:C injection (early gestation). All other tissue collections were performed on E15.5 (mid-to-late gestation; total pregnancy duration in rats is ~22 days). Euthanasia was conducted using mild carbon dioxide asphyxiation, followed by thoracotomy. For samples collected on E8.5, whole conceptuses were isolated and snap-frozen in liquid nitrogen. For tissues collected on E15.5, embryos were collected, and whole brains were removed and fixed in 4% paraformaldehyde for immunohistochemical analysis, or cortical tissue was minced and processed for NPC and neurosphere culture.

### RNA Sequencing

RNA was extracted from tissue by homogenizing in RiboZol (Amresco). The aqueous phase was diluted with 70% ethanol and placed on RNeasy columns (Qiagen), treated with DNase I, and purified. RNA integrity was assessed using an Agilent 2100 bioanalyzer (Agilent Technologies), and indexed libraries were generated following rRNA reduction. Libraries were then sequenced with NextSeq High Output 75 cycle sequencing kit (Illumina) at the London Regional Genomics Center. Reads from ^*^.fastq files were aligned to the rat reference genome (Rnor_6.0.91) using CLCBio Genomics Workbench (Qiagen version 10.1.2), and transcript abundance was expressed as reads per kilobase of transcript per million mapped reads (RPKM). Analysis was restricted to transcripts in which average RPKM was >0.1 among either saline or PolyI:C-exposed conceptuses. Statistical significance was calculated by empirical analysis of digital gene expression, followed by Bonferroni's correction. Data are available on the Gene Expression Omnibus (GSE145167). Gene ontology pathway analysis was completed using DAVID Functional Annotation Bioinformatics (19).

### Quantitative RT-PCR

RNA was extracted from cells and tissues by lysing in RiboZol (Amresco) as directed by the manufacturer. Complementary DNA was made from purified RNA using High Capacity complementary DNA Reverse Transcription kit (ThermoFisher Scientific), diluted 1:10, and used for quantitative RT-PCR. Complementary DNA was mixed with SensiFAST SYBR green PCR Master Mix (FroggaBio) and primers (Table 1). Amplification and fluorescence detection were conducted using a CFX Connect Real-Time PCR system (Bio-Rad Laboratories). Cycling conditions included an initial holding step (95°C for 3 min), followed by 40 cycles of two-step PCR (95°C for 10 s, 60°C for 45 s), and a dissociation step (65°C for 5 s, and a sequential increase to 95°C). The comparative cycle threshold (ΔΔCt) method was used to calculate relative mRNA expression, using the geometric mean of Ct values obtained from amplification of five genes (*Rn18s, Ywhaz, Eef2, Gapdh*, and *Actb*) as reference RNA.

**Table 1:** List of primers used for RT-PCR.

**Gene**	**Accession number**	**Forward**	**Reverse**	**Size (kB)******
*Actb*	NM_031144.3	5′-AGCCATGTACGTAGCCATCC-3′	5′-CTCTCAGCTGTGGTGGTGAA-3′	227
*Ccl5*	NM_031116.3	5′-CCTTGCAGTCGTCTTTGTCA-3′	5′-GAGTAGGGGGTTGCTCAGTG-3′	174
*Cxcl10*	NM_139089.1	5′-TGTCCGCATGTTGAGATCAT-3′	5′-GGGTAAAGGGAGGTGGAGAG-3′	203
*Dll1*	NM_032063.2	5′-GCACGAGAAAACCAGAAAGC-3′	5′-TCTTCAAAGACCCAGGGATG-3′	239
*Dll3*	XM_006228611.3	5′-GACTCACAGCGCTTCCTTCT-3′	5′-TCTTCGGGATGATTCCAGTC-3′	244
*Dll4*	NM_001107760.1	5′-ACCTTTGGCAATGTCTCCAC-3′	5′-TTGGATGATGATTTGGCTGA-3′	215
*Doc2b*	NM_031142.1	5′-GAGCCAGCAAGGCAAATAAG-3′	5′-GTGTGGTTGGGTTTCAGCTT-3′	210
*Eef2*	NM_017245.2	5′-CGCTTCTATGCCTTCGGTAG-3′	5′-GTAGTGATGGTGCCGGTCTT-3′	235
*Gapdh*	NM_017008.4	5′-AGACAGCCGCATCTTCTTGT-3′	5′-CTTGCCGTGGGTAGAGTCAT-3′	206
*Il1b*	NM_031512.2	5′-AGGCTTCCTTGTGCAAGTGT-3′	5′-TGAGTGACACTGCCTTCCTG-3′	229
*Jag1*	NM_019147.1	5′-ATCGCATCGTACTGCCTTTC-3′	5′-GGCAATCCCTGTGTTCTGTT-3′	178
*Jag2*	NM_001375303.1	5′-TGCACTTTAACCGTGACCAA-3′	5′-AAAGACACAGCCACCTCCAC-3′	165
*Notch1*	NM_001105721.1	5′-GTTTGTGCAAGGATGGTGTG-3′	5′-CCTTGAGGCATAAGCAGAGG-3′	161
*Notch2*	NM_024358.2	5′-TCAATCGCTTCCAGTGTCTG-3′	5′-TGCAGATGCAGGTGTAGGAG-3′	248
*Notch3*	NM_020087.2	5′-CCCACAAGCCCTGTAGTCAT-3′	5′-TCACATTGATGACCCTGGAA-3′	210
*Notch4*	NM_001002827.1	5′-CACCTGCCTGAGGCTATCTC-3′	5′-GAGCTCTTCCAGAGGGCTTT-3′	246
*Rn18s*	NM_046237.1	5′-GCAATTATTCCCCATGAACG-3′	5′-GGCCTCACTAAACCATCCAA-3′	137
*Rnf112*	NM_138613.1	5′-CGCCAAGAAGGAGTTTGAAG-3′	5′-CAGAAGCGCATTGTGTAGGA-3′	242
*Rsad2*	NM_138881.1	5′-GGGATGCTAGTGCCTACTGC-3′	5′-CTGAGTCTCCTTGGGCTCAC-3′	173
*Sema5c*	NM_017308.1	5′-ACTGTCTTTCCCCCTGTCCT-3′	5′-GTGGGGGCTCCTACACAGTA-3′	240
*Ywhaz*	NM_013011.3	5′-TTGAGCAGAAGACGGAAGGT-3′	5′-CCTCAGCCAAGTAGCGGTAG-3′	200

*kB, Kilobases*.

### Isolation of NPCs and Neurosphere Culture

Single-cell suspensions of cortical cells can be isolated from the embryonic telencephalon and propagated as spherical NPC-containing masses called neurospheres (20). NPC culture was based on the method described by Ghosh et al., with modifications (21). Embryos were collected on E15.5, and DNA isolated from tails using reagents from REDExtract-N-Amp DNA isolation kit (Sigma-Aldrich) to evaluate sex of embryos, based on presence or absence of the *Sry* gene. Primers used for *Sry* genotyping were 5′-TGGGAATGTATGCTGGCATA-3′ and 5′-CCTCTCATGCCCAGAGTGAC-3′. Using a dissecting microscope, skin, skull, and meninges were removed to access the cortex, then cortical tissue was removed and placed in a dish containing fresh primary culture media (NeuroCult Proliferation Kit, Stem Cell Technologies). Cells were gently dissociated, plated at a density of 120,000 cells/ml, and incubated at 37°C and 5% CO_2_ initially for 4 days. Under these conditions, cells efficiently proliferated to generate neurospheres. To determine their proliferation capacity, after 3 days in culture, neurospheres were treated with 10 mM 5-ethynyl-2′-deoxyuridine (EdU) for 24 h, followed by immunohistochemistry as described below.

After 4 days, primary cortical NPCs were isolated from neurospheres. In brief, neurospheres were mechanically dissociated, centrifuged, and resuspended in either proliferation or differentiation medium. Cell viability was determined through trypan blue exclusion, and 25,000 viable cells/ml were distributed into 24-well plates containing sterile coverslips precoated with poly-D lysine (10 μg/ml) and laminin (10 μg/ml; both from Sigma-Aldrich). For experiments in which male and female embryos were individually analyzed, cells from each embryo were cultured in their own well until genotyping was performed, then male or female cells were combined into a single well. For all other experiments, cells from 3-5 embryos were combined. Cells plated for proliferation experiments received NeuroCult proliferation medium, and cells plated for differentiation experiments were cultured in NeuroCult differentiation medium (both from Stem Cell Technologies). Cells were cultured for 3 days prior to treatment with 10 mM EdU for 24 h, followed by immunofluorescence as described below.

### Immunohistochemistry

Whole fetal brains were collected on E15.5 and placed in 4% paraformaldehyde overnight. Brains were transferred to 30% sucrose for at least 24 h, and then embedded in O.C.T. compound and stored at −80°C until sectioning. Coronal cryosections were obtained at 12 μm thickness. For neurosphere cryosectioning, media were carefully removed from neurospheres after allowing them to settle to the bottom of a conical centrifuge tube, and then 4% paraformaldehyde was added to the tube. Neurospheres were transferred to 30% sucrose for at least 24 h, embedded in O.C.T. compound, and cryosectioned at 10 μm thickness.

Sections of fetal brain and neurospheres were post-fixed for 10 min in 4% paraformaldehyde, permeabilized in PBS containing 1% bovine serum albumin and 0.3% Triton-X, and blocked using 10% normal goat serum to reduce non-specific antibody binding. Sections were immersed in primary antibodies as described below. The following day, species-appropriate, fluorescent-conjugated secondary antibodies were applied for 1 h, followed by Hoechst nuclear stain (ThermoFisher Scientific). For detection of EdU, sections of EdU-treated neurospheres were probed with an Alexa488-conjugated EdU monoclonal antibody (ClickIt EdU proliferation kit, ThermoFisher Scientific), followed by Hoechst nuclear stain. Sections were then mounted using Fluoromount G (SouthernBiotech), and fluorescence detected using a Nikon ECLIPSE Ni series microscope equipped with a Ds-Qi2 camera. Confocal microscopy was performed using a Zeiss LSM800 series confocal laser scanning microscope.

### Immunofluorescence

Cell culture media were removed, and cells fixed in 4% paraformaldehyde. Detection of EdU-positive cells was performed using an Alexa488-conjugated EdU monoclonal antibody. Nuclei were subsequently counterstained using Hoechst nuclear stain. Coverslips were then mounted, and fluorescence detected as described above. The percentage of EdU-positive cells was calculated by dividing the number of EdU-positive cells by the total number of cells (as determined by detection of nuclei by Hoechst staining), multiplied by 100.

For detection of various proteins, antibodies targeting Sox2 (1:100, 48-1400, ThermoFisher Scientific), βIII-Tubulin (β3T; 1:1000, 60052, Stem Cell Technologies), Pax6 (1:250, 42-6600, ThermoFisher Scientific), NeuN (1:200, MAB377, EMD Millipore), Phospho-Histone H3 (1:800, 3377, Cell Signaling Technology), GFAP (1:200, 12389, Cell Signaling Technology), and Notch1 (1:200, 4380, Cell Signaling Technology) were diluted in PBS and applied overnight at 4°C. The following day, species-appropriate, fluorescent-conjugated secondary antibodies (AlexaFluor anti-mouse 488 and anti-rabbit 555, ThermoFisher Scientific) were applied for 1 h, followed by Hoechst nuclear stain (ThermoFisher Scientific). Coverslips were then mounted, and fluorescence detected as described above.

### Western Blotting

Protein expression was evaluated by western blotting. Total protein was isolated by immersing cells in radioimmunoprecipitation assay lysis buffer (50 mM Tris, 150 mM NaCl, 1% NP40, 0.5% sodium deoxycholate, 0.1% SDS, pH 7.2) supplemented with protease inhibitor cocktail (Sigma-Aldrich). Nuclear and cytoplasmic lysates were prepared using the NE-PER kit (ThermoFisher Scientific). A modified bicinchoninic acid assay (Bio-Rad Laboratories) was used to measure total protein concentrations. Approximately 10 μg of cell lysates were mixed with 4× sample loading buffer (final concentration: 62.5 mM Tris pH 6.8, 2% SDS, 10% glycerol, 0.025% bromophenol blue, 50 mM dithiothreitol), boiled for 10 min, and subjected to SDS-polyacrylamide gel electrophoresis. Proteins were then transferred to polyvinylidene difluoride membranes, blocked with 5% bovine serum albumin in TBS containing 0.1% Tween-20, and probed with primary antibodies specific for phospho-histone H3 (1:1000, 3377, Cell Signaling Technology), Notch1 (1:1000, 4380, Cell Signaling Technology), Histone H3 (1:1000, 4499, Cell Signaling Technology), and β-actin (1:500, sc-47778, Santa Cruz Biotechnology). Following incubation with species-appropriate, infrared-conjugated secondary antibodies (Cell Signaling Technology), protein signals were detected using a LI-COR Odyssey imaging system (LI-COR Biosciences).

### Statistical Analysis

Statistical comparisons between two means were conducted using unpaired, two-tailed Student's *t*-tests, whereas statistical comparisons of three or more means were conducted using analysis of variance, followed by Tukey's *post-hoc* test. Sex-dependent effects were compared using two-way analysis of variance followed by Sidak's multiple comparison. Assessment of changes in transcript abundance was conducted using nine conceptuses collected from three dams per group. Data were independently validated with an additional nine conceptuses from three dams per group. Fetal cortical thickness was analyzed by obtaining images of the cortex from three distinct regions (anterior, middle, posterior) separated from each other by ~100 μm, using at least 10 male and 10 female brains collected from a total of three different litters per group. Total cortical thickness, as well as thickness of each layer (Sox2+, β3T+), was measured in three independent areas of each image using ImageJ (22). Data are presented as individual length measurements in μm ± SEM. For comparison of proliferation capacity between neurospheres isolated from saline and PolyI:C-treated dams, at least 1,655 cells per dam prepared from four different litters per treatment were used for analyses. Data are presented as percent EdU-positive cells per neurosphere ± SEM. For comparing proliferation capacity in NPCs following disaggregation, five fields of view were randomly imaged across two technical replicates, and cell numbers were determined using an in-house automated cell-counting program (MATLAB) to obtain total cell counts (based on the number of Hoechst-positive nuclei) and EdU-positive cells. At least 300 cells per treatment obtained from 3 dams per group were counted. The percent EdU-positive cells in each image was then obtained. All differences were considered statistically significant when *P* < 0.05. Graphing and statistical analyses were performed using GraphPad Prism 7.0.

## Results

### Maternal PolyI:C Exposure During Early Pregnancy Induces an Antiviral Response in the Conceptus and Alters Expression of Genes Associated With Nervous System Development

**Figure 1:**
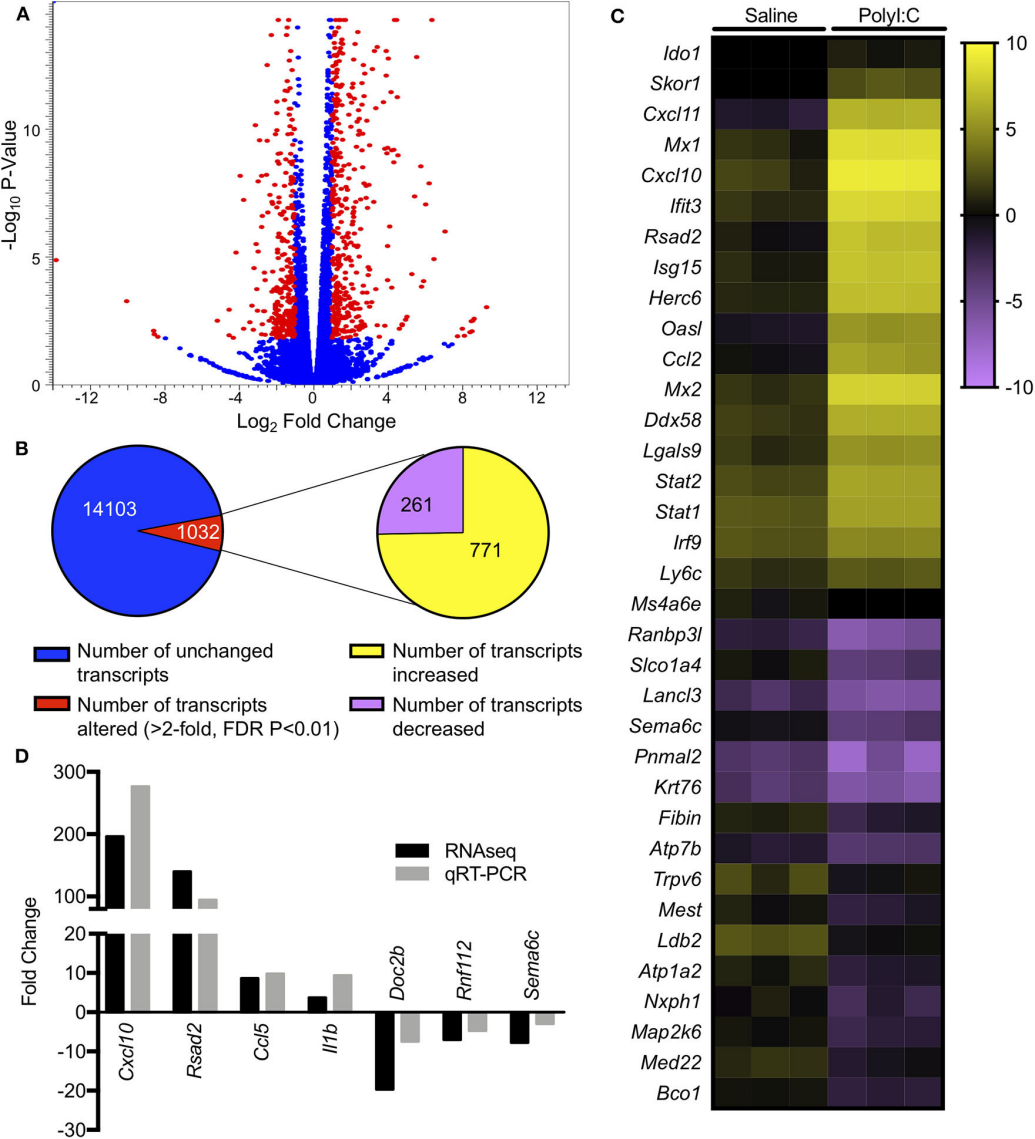
Gene expression changes in the conceptus following maternal exposure to PolyI:C. **(A)** Volcano plot depicting number of unique transcripts expressed in the conceptus following PolyI:C exposure relative to conceptuses exposed to saline. The x-axis represents magnitude of fold-change (log_2_), and the y-axis shows *P*-value (log_10_). Transcripts altered 2-fold or more (false discovery rate *P* < 0.01) following PolyI:C are shown in red. **(B)** Pie chart showing number of transcripts upregulated and downregulated in conceptuses exposed to PolyI:C compared to those exposed to saline. **(C)** Heat map showing RPKM values (log_2_) of select transcripts in conceptuses following maternal exposure to saline or PolyI:C. **(D)** Quantitative RT-PCR validation of select transcripts in relation to values obtained using RNA-Seq. Data are normalized to values obtained from saline-exposed conceptuses. All data are based on lysates from three conceptuses pooled from each of three dams (nine conceptuses total) per treatment.

PolyI:C is a synthetic analog of dsRNA and has been used to stimulate cellular antiviral responses *in lieu* of an active pathogen. In rodents (10), and primates (23), exposure of dams to PolyI:C during early or mid-pregnancy elicits long-term cognitive and behavioral deficits in offspring, suggesting that PolyI:C, or the antiviral response incited by PolyI:C, interferes with normal brain developmental processes. Transcriptome profiles have been generated using neonatal or postnatal brain tissue, days or weeks following prenatal exposure to PolyI:C (24, 25). However, acute gene expression changes in the conceptus (encompassing decidua, embryo, and extraembryonic tissues) triggered by PolyI:C have not been extensively characterized. Therefore, our first goal was to use RNASeq to profile global gene expression changes in the conceptus following acute PolyI:C treatment. Overall, from a total of 15,135 transcripts examined, exposure of dams to PolyI:C induced significant changes in 1,032 transcripts in the conceptus (≥2-fold increased or decreased compared to dams injected with saline, FDR *P* < 0.01). Of these, 771 genes were upregulated, and 261 genes were downregulated (Figure 1). The 20 transcripts exhibiting the highest increase and decrease following maternal PolyI:C exposure are listed in Tables 2, 3, respectively. Among upregulated transcripts, maternal PolyI:C exposure induced expression of interferon-stimulated genes (e.g., *Isg15*, 101.5-fold; *Ifit3*, 140.5-fold; *Mx1*, 196.8-fold; *Mx2*, 108.2-fold), anti-viral molecules (e.g., *Rsad2*, 139.9-fold; *Stat1*, 9.5-fold; *Stat2*, 15.1-fold), and inflammatory chemokines (e.g., *Cxcl10*, 196.3-fold; *Cxcl11*, 255.7-fold; *Ccl2*, 65.9-fold; Figure 1C, all FDR *P* < 0.01). Interestingly, a considerable number of transcripts that were decreased after PolyI:C exposure are associated with neurulation and neural development (e.g., *Ranbp1*, 13.4-fold; *Sema6c*, 7.8-fold; *Pnmal2*, 12.1-fold; *Nxph1*, 4.5-fold; Figure 1C, all FDR *P* < 0.01). To validate results obtained from RNASeq on a separate cohort of samples, quantitative RT-PCR was conducted on conceptuses exposed to saline or PolyI:C. Expression of *Cxcl10, Rsad2, Ccl5*, and *Il1b* was increased in conceptuses following maternal PolyI:C exposure, and expression of *Doc2b, Rnf112*, and *Sema6c* was decreased, with fold changes consistent with results obtained using RNASeq (Figure 1D).

**Table 2:** List of top 20 increased genes following maternal PolyI:C exposure.

**Gene**	**Fold change**	**Saline RPKM**	**PolyI:C RPKM^†^**
*Ido1*	636.88	0	0.68
*Skor1*	398.92	0	0.18
*Defb52*	378.92	0	1.30
*Tnf*	364.57	0	0.29
*RGD1305184*	280.10	0.18	44.85
*Cxcl11*	255.69	0.41	91.90
*Iqcf3*	255.49	0	0.26
*Gbp3*	212.40	0.02	4.65
*Mx1*	196.83	2.06	358.59
*Cxcl10*	196.34	3.12	541.00
*Gbp1*	194.82	0.22	38.16
*Ubp*	38.16	0.86	142.26
*Ifit3*	140.50	2.35	291.69
*Rsad2*	139.88	1.13	137.84
*Olr1735*	134.79	0	0.68
*Mx2*	108.19	2.58	246.85
*Isg15*	101.53	1.71	154.19
*Usp18*	97.96	0.64	55.25
*Cxcl9*	96.43	2.84	243.99
*Olr1734*	89.75	0	0.42

† *PolyI:C, Polyinosinic:Polycytidylic acid; RPKM, Reads Per Kilobase of transcript per Million mapped reads*.

**Table 3:** List of top 20 decreased genes following maternal PolyI:C exposure.

**Gene**	**Fold change**	**Saline RPKM**	**PolyI:C RPKM^†^**
*Ms4a6e*	−1046.99	1.28	0
*LOC103690002*	−15.47	2.84	0.16
*Slco1a4*	−13.43	1.30	0.09
*Ddo*	−13.43	0.12	0.01
*RGD1561034*	−9.23	0.87	0.01
*Grcc10*	−8.79	4.54	0.47
*Clec4a*	−8.52	0.43	0.04
*Lancl3*	−7.83	0.68	0.08
*Sema6c*	−7.76	0.16	0.02
*Btnl9*	−7.45	0.72	0.08
*Myom2*	−6.93	0.21	0.03
*Muc16*	−6.88	0.16	0.02
*Abcg4*	−6.77	0.61	0.08
*Gria2*	−6.71	0.63	0.08
*Shc3*	−6.25	0.32	0.05
*Kcng1*	−6.16	0.13	0.02
*Scn7a*	−5.81	0.39	0.06
*Ldb2*	−5.72	5.73	0.89
*Etv1*	−5.67	0.29	0.05
*Rbp7*	−5.57	2.37	0.38

† *PolyI:C, Polyinosinic:Polycytidylic acid; RPKM, Reads Per Kilobase of transcript per Million mapped reads*.

**Figure 2:**
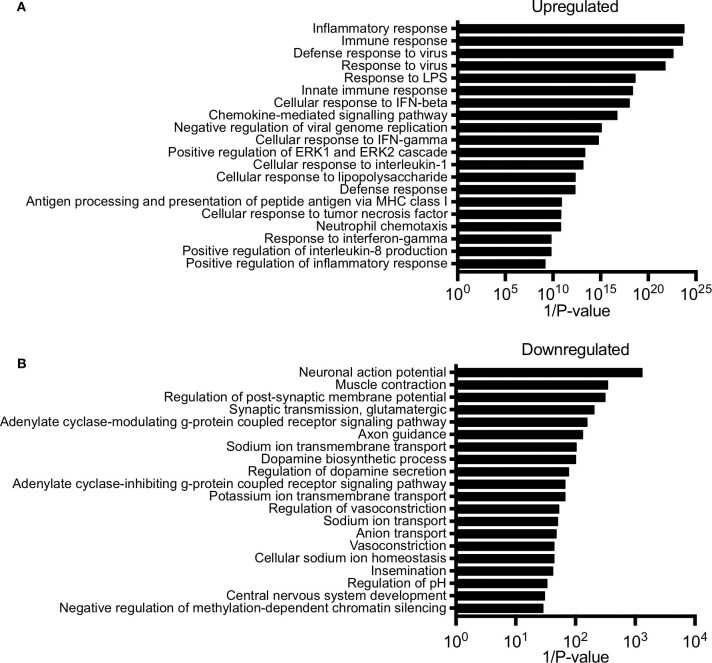
Pathway analysis of unique gene signatures upregulated and downregulated in the conceptus following maternal exposure to PolyI:C. **(A)** Top 20 gene pathways upregulated in the conceptus following administration of PolyI:C to pregnant dams. **(B)** Top 20 gene pathways downregulated in the conceptus following administration of PolyI:C to pregnant dams. Pathway analysis was conducted by inputting genes changed more than 2-fold compared to conceptuses collected following maternal exposure to saline, and with a false discovery rate < 0.01.

Next, we performed gene ontology analysis to identify pathways that were significantly altered in the conceptus following maternal PolyI:C exposure. Not surprisingly, pathways that were upregulated after PolyI:C exposure included those associated with antiviral pathways and inflammation. Examples of upregulated pathways include: negative regulation of viral replication pathway (*P* = 8.2E-16, 17 genes), innate immune response (*P* = 4.5E-19, 47 genes), and inflammatory response (*P* = 1.8E-24, 57 genes, Figure 2A). Intriguingly, pathways associated with decreased gene expression were related to nervous system function and development, including central nervous system development (*P* = 3.3E-02, 5 genes); axon guidance (*P* = 7.6E-03, 7 genes); and neuronal action potential (*P* = 7.7E-04, 5 genes, Figure 2B). Thus, maternal PolyI:C exposure during early pregnancy induces robust transcriptome changes in the conceptus, including increased expression of genes associated with antiviral and inflammatory pathways and reduced expression of genes associated with neurological development.

### Maternal PolyI:C Exposure During Early Pregnancy Alters Cortical Architecture in the Fetus

**Figure 3:**
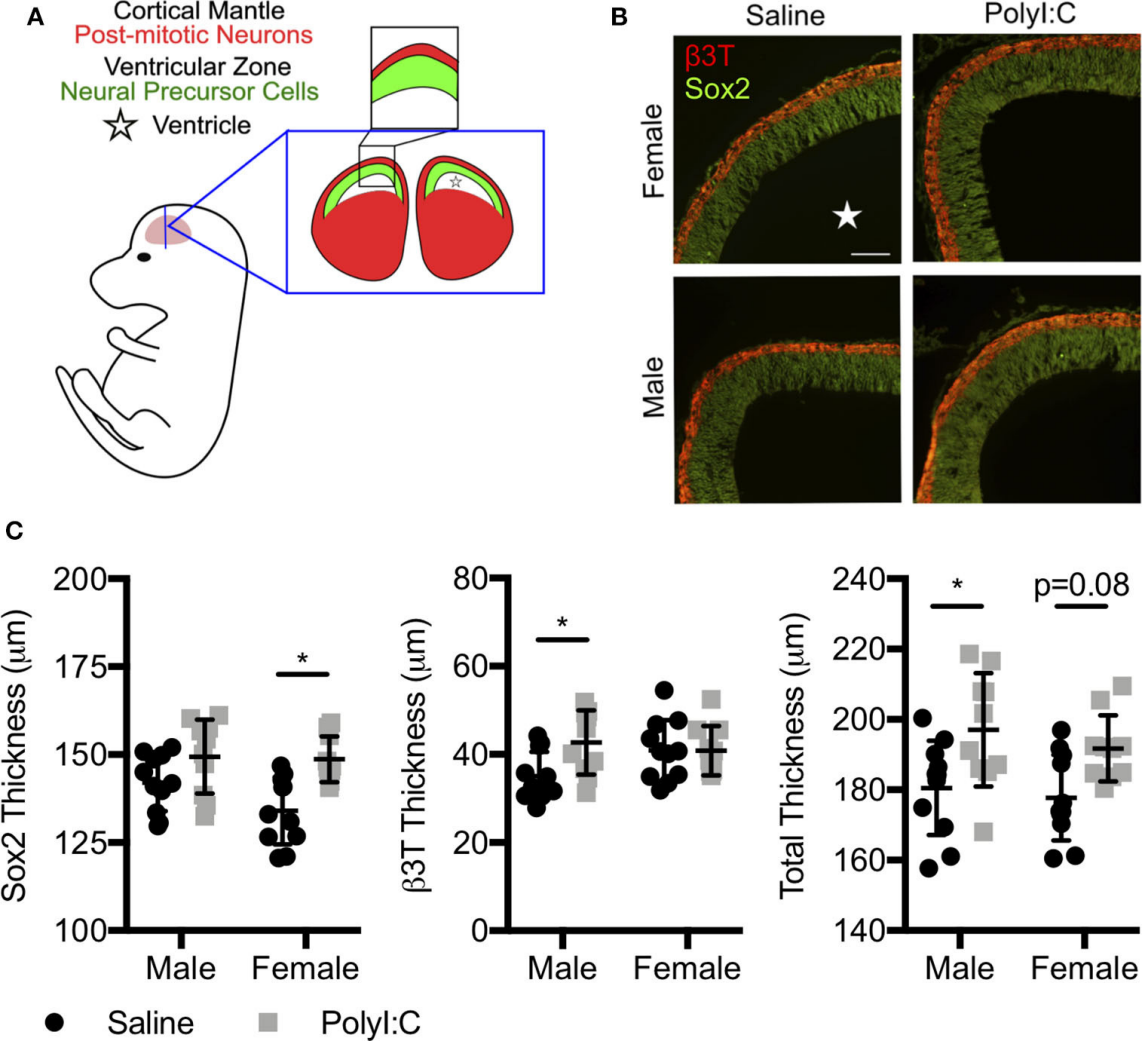
Prenatal exposure to PolyI:C prompts changes in cortical architecture. Dams were injected with either saline or PolyI:C on E8.5, and whole fetal brains were collected 1 week later. **(A)** Schematic depiction of the layers of the rat fetal cortex at E15.5. **(B)** Coronal sections of the cortex at E15.5 were stained for β3T (red) and Sox2 (green) to delineate the CM and VZ, respectively. **(C)** Measurements showing total cortical thickness and thickness of the CM and VZ in male and female fetuses. Graphs represent means ± SEM. A star () denotes the ventricle. Statistical analyses were performed using two-way analysis of variance and Sidak's multiple comparison. Data are represented as mean ± SEM and asterisks denote statistical significance (*P < 0.05; N ≥ 10 fetuses per sex from at least three dams per group). Scale bar = 100 μm.

Since offspring born to dams exposed to PolyI:C during early pregnancy are predisposed to neurobehavioral impairments, and we found decreased expression of various genes associated with central nervous system development following maternal PolyI:C exposure, we sought to elucidate whether cortical architecture was altered in developing fetal brains. Cortical neurogenesis begins around E11 in rats, peaks at E15, and slows by E17 (26). Consequently, we measured cortical thickness, including thickness of the cortical mantle (CM; delineated using the neuronal marker β3T) and ventricular zone (VZ; identified using Sox2, which is highly expressed in NPCs, Figures 3A,B) in coronal sections of fetal brains on E15.5, 1 week following maternal saline or PolyI:C treatment. Male fetuses prenatally challenged with PolyI:C showed an 8.3% increase in total cortical thickness on E15.5 compared to saline-exposed male fetuses (Figure 3C, *P* < 0.05). The increased cortical thickness in male fetuses was primarily attributed to a thicker CM (20% increase in PolyI:C-exposed fetuses compared to saline, Figure 3C, *P* < 0.05). The VZ also appeared to be increased in PolyI:C-exposed male fetuses, but it was not statistically significant. Interestingly, thickness of the VZ in female fetuses prenatally challenged with PolyI:C was increased by 10% (Figure 3C, *P* < 0.05), whereas there was no change in thickness of the CM. Total cortical thickness appeared to be thicker in females exposed to PolyI:C, but did not reach statistical significance (*P* = 0.08). Our results indicate that maternal PolyI:C exposure during early pregnancy caused sex-specific alterations in cortical architecture at mid-to-late gestation, and in general was associated with increased thickness of the developing cerebral cortex.

### Increased Proliferation of Fetal NPCs Following Maternal Exposure to PolyI:C

**Figure 4:**
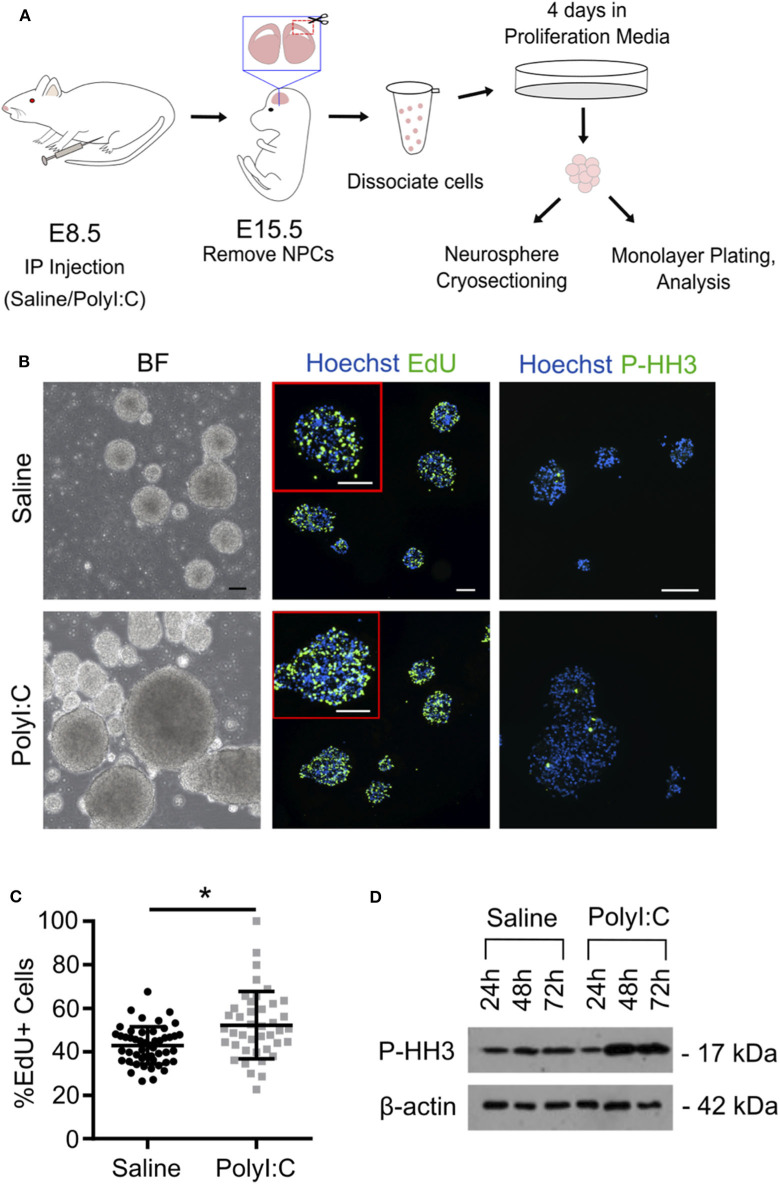
Maternal exposure to PolyI:C increases proliferation potential of neurospheres prepared from fetal cortices. Pregnant rats were administered saline or PolyI:C on E8.5, and NPCs were isolated from E15.5 fetal cortices for neurosphere culture. **(A)** Schematic depiction of experimental protocol. **(B)** The two left panels show neurospheres produced from cortical NPCs imaged using brightfield. The middle two panels show EdU incorporation in cryosectioned neurospheres. The right two panels show immunofluorescence for phospho-histone H3 (P-HH3). In the middle and right panels, Hoechst is used to counterstain nuclei. Scale bar = 100 μm. **(C)** Percent EdU positive cells in each neurosphere were quantified from each treatment group. **(D)** Western blot analysis of P-HH3 expression in neurospheres following 24, 48, and 72 h culture. β-actin was used as a loading control. Statistical analyses were performed using Student's *t*-test. Data are represented as mean ± SEM. Data significantly different (*P* < 0.05) from controls are indicated by an asterisk (*5–6 fetuses from each of 3 dams per treatment were used for neurosphere generation).

Our results indicate that maternal PolyI:C treatment at early gestation results in altered thickness of cortical layers in fetuses. This might be mediated through abnormal functioning of NSCs/NPCs. Thus, for our next series of experiments, pregnant dams were administered saline or PolyI:C on E8.5, and NPCs were isolated from fetal cortices on E15.5 (Figure 4A). Cells isolated from fetal cortices expressed Pax6, Nestin, and Sox2, and displayed morphology consistent with NPCs, including formation into neurospheres within 4 days, at which point they were either fixed and cryosectioned, or disaggregated and plated into monolayers. Interestingly, neurospheres isolated from fetuses exposed prenatally to PolyI:C showed rapid growth and formed into larger colonies, suggesting that they may have a higher proliferative index compared to neurospheres isolated from fetuses collected from saline-treated dams (Figure 4B). To examine the proliferative capacity of NPCs, we measured the percentage of cells within neurospheres that incorporated EdU, and found that neurospheres prepared from fetal cortical tissue collected from PolyI:C-treated dams exhibited a 9.3% increase in EdU incorporation compared to neurospheres prepared from saline-exposed fetuses (Figure 4C, *P* < 0.05). Furthermore, neurospheres from PolyI:C-treated pregnancies had increased staining for phospho-histone H3 (P-HH3), a marker of mitotically-active cells, as shown using both immunofluorescence (Figure 4B) and western blotting (Figure 4D).

**Figure 5:**
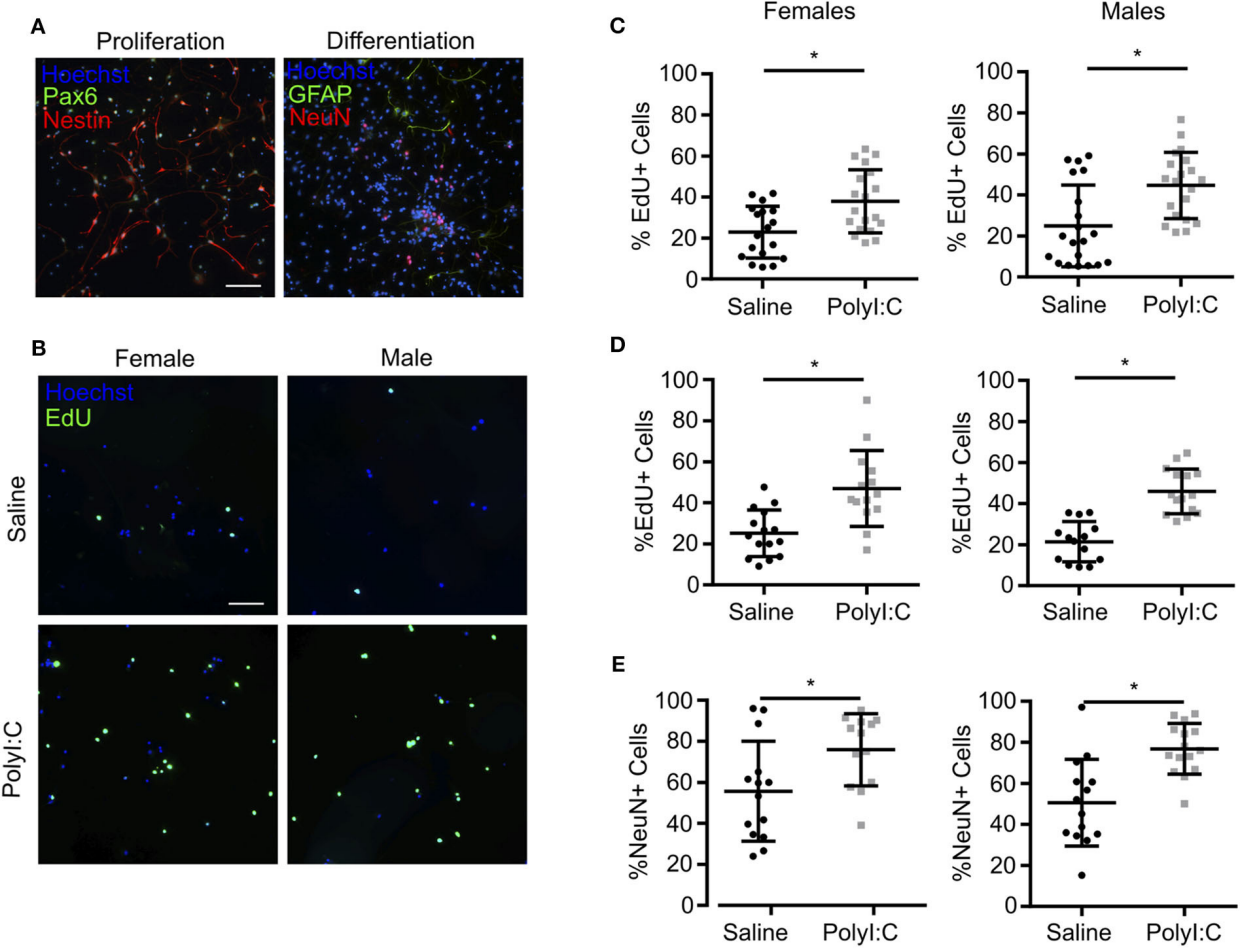
NPCs isolated from fetal cortices challenged prenatally with PolyI:C exhibit increased proliferation potential. Pregnant rats were administered saline or PolyI:C on E8.5, and NPCs were isolated from E15.5 cortices for neurosphere culture. Neurospheres from male or female embryos were pooled, and then mechanically dissociated to form monolayers. Monolayers were subsequently cultured in media to promote stem or differentiated states. **(A)** Representative images of NPC monolayers cultured in proliferation conditions stained for Pax6 (green) and Nestin (red), or cultured in differentiation conditions stained for GFAP (green) and NeuN (red). Hoechst (blue) was used to counterstain nuclei. **(B)** Representative images of EdU incorporation in NPCs prepared in proliferation conditions from male and female cortices challenged prenatally with saline or PolyI:C 1 week prior. **(C)** Percentage of male and female NPCs that incorporated EdU during culture in proliferation conditions. **(D)** Percentage of male and female NPCs maintained in differentiation media that incorporated EdU. **(E)** Percentage of male and female NPCs maintained in differentiation media immunoreactive for NeuN indicating their differentiation capacity. Statistical analyses were performed using Student's *t*-test. Data are represented as mean ± SEM. Data significantly different (*P* < 0.05) from controls are indicated by an asterisk (*2–3 fetuses per sex collected from at least 3 dams). Scale bar = 50 μm.

When neurospheres are disaggregated and cells plated as monolayers, NPCs can be maintained as a proliferative population expressing Nestin, Pax6, and Sox2, or induced to differentiate into NeuN+ neurons and GFAP+ glia (Figure 5A and Supplemental Figure 1). Thus, to determine the proliferative capacity of NPCs using monolayer cultures, neurospheres prepared from individual fetuses were disaggregated, sex determined through *Sry* genotyping, and male or female NPCs combined and re-plated as monolayers. In NPCs prepared from E15.5 fetal brains 1-week following maternal PolyI:C exposure, ~15% more NPCs incorporated EdU regardless of whether they were derived from a male or female fetus compared to NPCs from saline-exposed fetuses (Figures 5B,C, *P* < 0.05), which is consistent with the increased proliferation of NPCs in neurospheres and thicker cortical layers in fetal brains prenatally challenged with PolyI:C (Figures 3, 4). The increased EdU incorporation was also evident if cells were cultured in conditions that favor differentiation (Figure 5D, *P* < 0.05). Additionally, we used NeuN to identify cells that underwent differentiation into neurons. In NPCs prepared from fetal cortices 1 week following maternal PolyI:C exposure, there was a 26.3% (*P* = 0.0003) and 20.2% (*P* = 0.0212) increase in NeuN-labeled cells in male and female NPCs, respectively (Figure 5E). Although both male and female NPCs prepared from fetuses exposed to PolyI:C have a higher differentiation potential compared to fetuses exposed to saline, the effect was more robust in male fetuses. Collectively, these results show that exposing pregnant rats to MIA during early pregnancy disrupts the normal proliferation potential and behavior of fetal NPCs.

### Altered Expression of Components of the Notch Signaling Pathway in Conceptuses Following Maternal Exposure to PolyI:C

**Figure 6:**
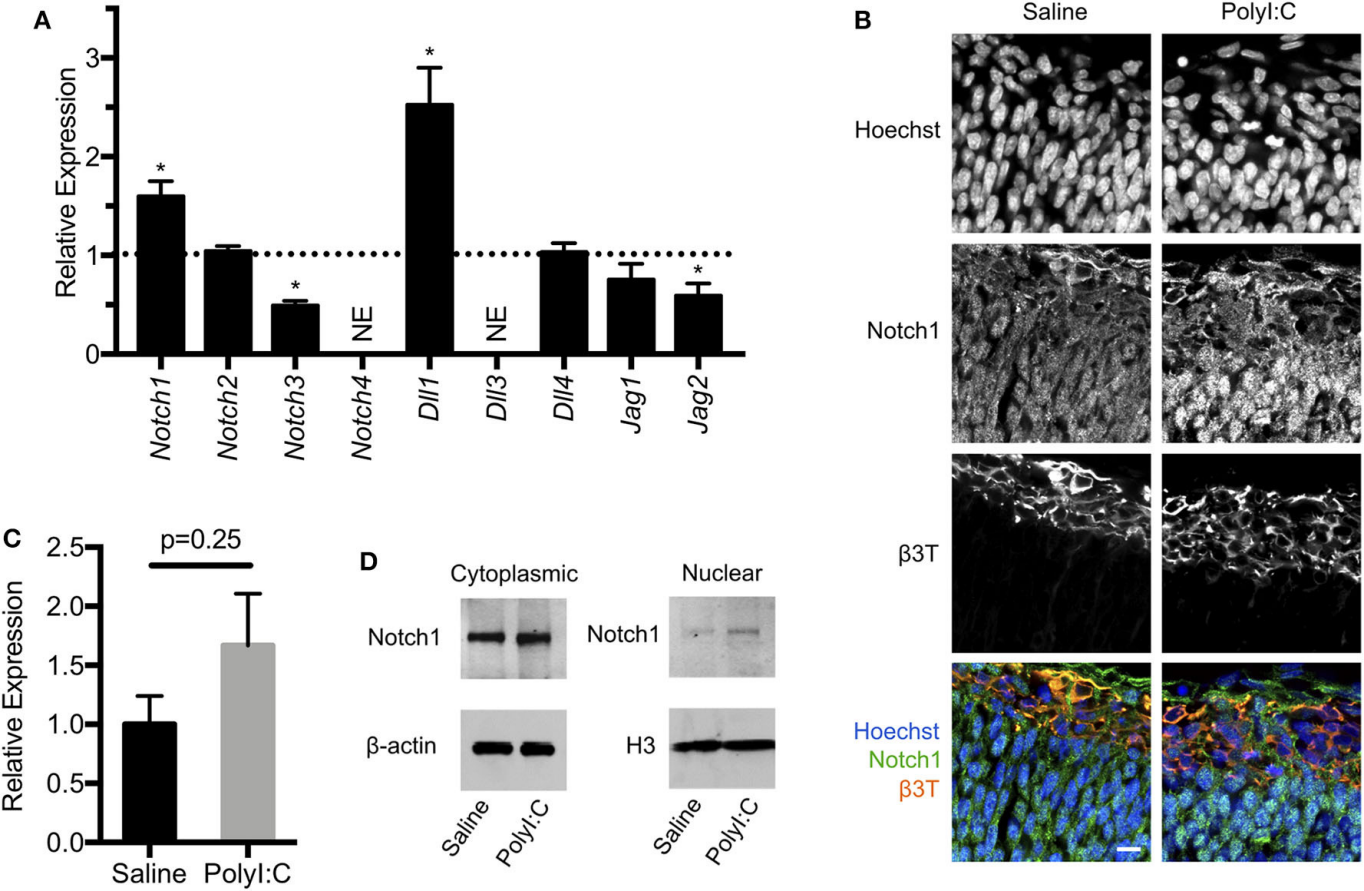
Maternal exposure to PolyI:C alters expression of Notch signaling components in the conceptus. **(A)** Transcript levels of Notch receptors (*Notch1-4*) and ligands (*Dll1, Dll3, Dll4, Jag1, Jag2*) in conceptuses 6 h following maternal exposure to PolyI:C. Transcript levels were normalized to levels in conceptuses from saline-exposed dams. NE, Not expressed (below threshold of detection). **(B)** Immunofluorescence showing Notch1 expression in E15.5 fetal cortices 1-week following maternal exposure to saline or PolyI:C. β3T was used to delineate the CM. Hoechst was used to stain nuclei. Note that Notch1 is expressed throughout the cortices, but qualitatively increased expression and nuclear accumulation of Notch1 is evident following prenatal challenge with PolyI:C. Scale bar = 10 μm. **(C)** Transcript levels of *Notch1* expression in neurospheres collected from cortices of E15.5 fetuses 6 h following maternal exposure to PolyI:C or saline. **(D)** Cytoplasmic and nuclear levels of Notch1 in NPCs collected from fetal cortices 1 week after maternal exposure to saline or PolyI:C. β-actin was used as a loading control for cytoplasmic lysates; histone H3 was used as a loading control for nuclear lysates. Statistical analyses were performed using Student's *t*-test. Data are represented as mean ± SEM. Data significantly different (*P* < 0.05) from controls are indicated by an asterisk (*3 fetuses collected from each of 3 dams per treatment).

The Notch signaling pathway is intricately involved in shaping the cell number and function of the vertebrate nervous system, and is well-known to facilitate binary cell fate choices, including the regulation of NPC maintenance and differentiation [reviewed in (27)]. In mammals, Notch signaling is controlled by cell-cell interactions, with Notch receptors (Notch1-4) on one cell activated by ligands [Delta-like (Dll)-1, Dll-3, Dll-4, Jagged (Jag)-1, and Jag-2] expressed on adjacent cells. Ligand-receptor interactions facilitate cleavage of the Notch intracellular domain, which translocates to the nucleus to activate target genes influencing cell fate in the nervous system. Since there are no intermediates between activation of Notch receptors in the cell membrane and their target site in the nucleus, Notch signaling is principally regulated by the expression of receptors, ligands, and enzymatic activity at the cell membrane. In our RNA-Seq analysis, we noted increased expression of *Notch1* (2.2-fold, FDR *P* < 0.01) and *Dll1* (4.72-fold, FDR *P* < 0.01) in conceptuses following MIA, which prompted us to evaluate expression of all receptors and ligands that participate in Notch signaling. Using quantitative RT-PCR, we confirmed that following prenatal challenge with PolyI:C, conceptuses exhibited increased expression of *Notch1* and *Dll1* compared to conceptuses exposed to saline, with fold-changes comparable to results obtained using RNA-Seq (Figure 6A, both *P* < 0.05). We also found reduced expression of *Notch3* and *Jag2* (51% decreased and 41% decreased, respectively, *P* < 0.05). Expression of *Notch3* and *Jag2* appeared to be similarly reduced in our RNA-Seq analysis, but the *P*-values did not reach our stringent cut-off (FDR *P*-value = 0.10 and 0.06, respectively). There was no change in *Notch2, Dll4*, or *Jag1* expression between saline or PolyI:C-exposed conceptuses, whereas *Notch4* and *Dll3* levels were below the threshold for detection (Cq>30 in both groups).

**Figure 7:**
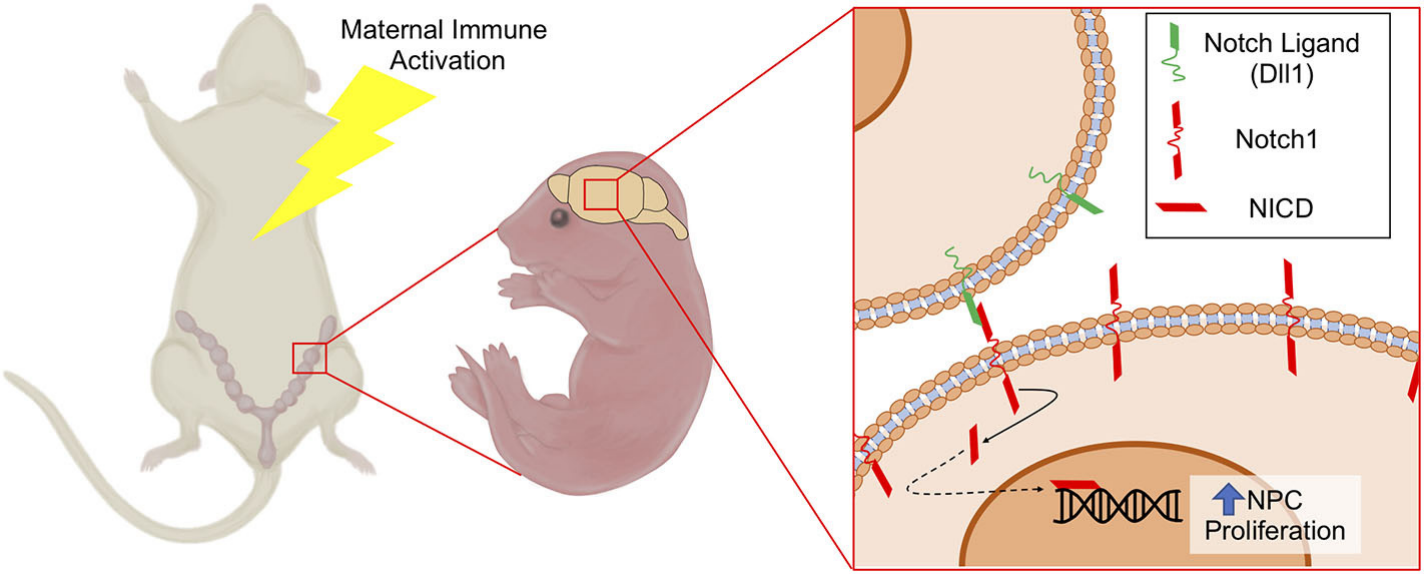
Schematic depicting increased NPC proliferation mediated by Notch1 signaling following MIA. MIA elicits increased expression of Notch1 and its ligand Dll1 in NPCs, which may contribute to altered NPC proliferation and differentiation dynamics leading to changes in cortical structure. NICD, Notch Intracellular Domain.

Notch1 is the prototypical Notch receptor implicated in maintaining NPC function. Since we found increased Notch1 expression in conceptuses following MIA, we sought to determine whether sustained Notch1 expression is evident in the developing fetal cortex, 1-week following maternal exposure to saline or PolyI:C. We found that Notch1 was expressed in cells throughout the cortex, with more intense immunoreactivity in nuclei located within the VZ in fetuses prenatally challenged with PolyI:C (Figure 6B). To determine whether increased Notch1 expression was evident in NPCs following MIA, we isolated NPCs from fetal cortices following maternal exposure to saline or PolyI:C 1 week earlier, and generated neurospheres. Although there was no change in *Notch1* mRNA expression in neurospheres collected from fetal brains 1-week following maternal exposure to PolyI:C, Notch1 protein was increased, particularly in nuclear lysates (Figures 6C,D). Collectively, our results indicate that maternal exposure to PolyI:C results in abnormalities in Notch signaling in the conceptus and fetal brain (schematically depicted in Figure 7).

## Discussion

Ample epidemiological and experimental evidence supports the notion that MIA during early pregnancy may interfere with the development of the brain leading to potential long-term neuropsychiatric and behavioral abnormalities. While many studies have focused on long-term changes on brain development and/or behavioral outcomes following MIA, molecular and structural changes in prenatal brain development are not clear, and such changes may be central for postnatal consequences. In this study, we identified significant changes to the transcriptome in conceptuses immediately following MIA in rats, including increased expression of genes involved in inflammatory and antiviral responses and suppression of genes involved in brain development. Increased thickness of the VZ was evident in female fetuses 1-week following MIA; total cortical thickness and thickness of the CM were increased in male fetuses. Furthermore, NPCs prepared from fetal cortices 1-week following MIA exhibited enhanced proliferation *in vitro*, which may contribute to increased thickness of the cerebral cortex and is likely mediated by altered Notch signaling. Together, these results suggest that MIA during early pregnancy causes rapid alterations in signaling pathways required for normal neurogenesis, leading to profound and persistent changes in cortical development.

Exposure of pregnant rodents or primates to the synthetic dsRNA mimetic PolyI:C is a well-established tool for modeling MIA (28). Many viruses produce dsRNA as part of their replication cycle. Eukaryotic cells have evolved the capacity to recognize dsRNA through at least three receptors: toll-like receptor 3 (TLR3), retinoic acid-inducible gene 1 (RIG1), and melanoma differentiation-associated protein-5 (MDA5), which instigate an antiviral response to combat viral infection and propagation (29). Since PolyI:C is sufficient to activate these receptors and trigger cellular antiviral responses, the use of PolyI:C provides a means to investigate the impact of MIA without confounding variability in the severity, spread, and duration of viral infections. Molecular and cellular analyses conducted on brains of adult offspring challenged prenatally with PolyI:C show profound and reproducible impairments that align with behavioral phenotypes of clear relevance to neuropsychiatric disorders including ASD, schizophrenia, and depression (12, 30). Moreover, behavioral abnormalities in offspring can be recapitulated by direct maternal treatment with PolyI:C-induced cytokines (e.g., IL-6), whereas blocking key pathways prevents neural and behavioral deficiencies following MIA (10, 31, 32). In our study, we exposed dams to PolyI:C during early gestation (E8.5), which is a sensitive timepoint for neurogenesis in rats, and can also indirectly impact brain development by disrupting formation and function of supporting organs like the placenta (16, 33). Although previous studies have analyzed the transcriptome of the fetal brain following maternal PolyI:C exposure (34), our study is the first to characterize global transcriptome changes in the whole conceptus (including the primordial nervous system and placenta as well as maternal decidua, which is a likely source of cytokines deleterious for brain development). A limitation of this approach was the inability to ascertain changes happening specifically in the nascent central nervous system following MIA, however, it did provide us the opportunity to identify molecules activated within the conceptus, and correlate these changes to pathways integral for brain development. In future studies, the use of single cell transcriptomics may facilitate identification of cell type-specific responses to MIA.

Our study revealed broad antiviral and inflammatory responses triggered within the conceptus rapidly following PolyI:C exposure. Among genes robustly upregulated following maternal PolyI:C exposure, those conventionally associated with MIA-associated neurological impairments (e.g., *Il6, Il1b*, and interferon-stimulated genes) were evident. A variety of other highly upregulated genes were identified in our analysis that may have important relevance for nervous system development and embryogenesis. For example, indoleamine 2,3-dioxygenase (*Ido1*) encodes a potent immunomodulatory protein that catabolizes tryptophan—a critical amino acid for synthesis of neuromodulators such as kynurenine and serotonin. High levels of IDO1 are associated with depressive behavior in rats as well as pain and depression in humans (35). Increased expression of the genes encoding C-X-C motif chemokine (CXCL)-10 and CXCL11 occurs during central nervous system inflammation (36). We also identified increased expression of a family of genes encoding interferon-induced transmembrane proteins, which restrict cellular viral entry and spread, but may exacerbate MIA and contribute to PolyI:C-induced embryonic lethality in mice (37). Thus, the conceptus competently produces a powerful immune response that may be required to combat viral infections, but this response may have consequences on processes essential for embryogenesis.

Previous studies have described an inverse relationship between inflammation and expression of genes associated with brain development and function. For example, a single intracerebroventricular injection of lipopolysaccharide into adult mice decreases expression of genes associated with memory and learning (e.g., e.g., *Egr1* and *Arc*) in the cortex (38). Systemic inflammation also reduces gene expression of cholinergic components within various brain structures (39), homeostatic genes in microglia (40), and pathways canonically associated with nervous system development and function, including genes penetrant to ASD (41, 42). Consistent with these studies, our transcriptomics analysis identified decreased expression of numerous genes including *Doc2b, Sema6c, Gria1, P2rx1, Scn10a*, and *Rnf112*, that are associated with neural induction, neurogenesis, and central nervous system development following maternal PolyI:C exposure. *Doc2b* encodes a protein that contributes to release of neurotransmitters and synaptic transmission (43). *Sema6c* encodes the transmembrane protein Semaphorin-6c, which plays an integral role in central nervous system connectivity and formation of the peripheral nervous system (44). Altered levels of glutamate receptor 1, encoded by *Gria1*, is a risk factor for schizophrenia (45). P2X purinoceptor 1, encoded by *P2rx1*, potentiates neurite outgrowth, while mutations in sodium voltage-gated channel alpha subunit 10, encoded by *Scn10a*, has been implicated in neurological disorders like multiple sclerosis and Pitt-Hopkins (46). RING finger protein 112, encoded by *Rnf112*, regulates neuronal differentiation during embryo development and maintains neural function in adults. Mice lacking *Rnf112* exhibit severe growth retardation and impaired neurocognitive development characterized by deficiencies in motor balance, learning, and memory (47). Collectively, the rapid repression of multiple genes critical for neurogenesis following MIA may interfere with the normal progression of nervous system development and predispose to long-term deficiencies in cognition and behavior. However, questions remain about specific neurogenic pathways in the primordial nervous system that are sensitive to MIA, and their relevance to long-term pathology in humans with neurobehavioral or cognitive impairments.

Since we found that MIA represses expression of many genes associated with nervous system development, we predicted that structural changes would be evident in the cerebral cortex—the area of the brain responsible for higher order thought, perception, and reason. Proper cortical development and expansion are dependent on coordinated regulation of NPC proliferation and cell fate specification; accumulating evidence indicates that these processes are disrupted following MIA. For example, MIA enhances expression of cell cycle-related genes and alters NPC proliferation patterns, resulting in increased cortical thickness and neuron density, brain overgrowth, regions of cortical dysplasia, and layering defects (32, 48–50). In this context, our findings of increased cortical thickness and enhanced NPC proliferation in fetuses 1-week following MIA support previous findings and suggest endogenous dysregulation of NPC self-renewal and differentiation. Although the thickness of the cerebral cortex in both male and female brains was increased by prenatal exposure to PolyI:C, structural changes exhibited a degree of sexual dimorphism, with males displaying a thicker CM and females a thicker VZ. In line with the increased CM thickness in male fetuses prenatally challenged with PolyI:C, NPCs isolated from male fetuses also had a more pronounced tendency to differentiate *in vitro*. The structural discrepancies between male and female brains following prenatal challenge with PolyI:C may reflect distinct responses to inflammation, including a heightened and prolonged surge of inflammatory cytokines such as IL-6 and IL-1β in male brains, and increased susceptibility to apoptosis in female brains, which could differentially predispose male and female offspring to distinct neuropsychiatric phenotypes (51, 52). Additionally, the duration of time in which NPCs exhibit dysregulated proliferation following maternal inflammation is not known. Whereas our study focused on changes occurring within 1 week of PolyI:C exposure, a transient surge of maternal IL-6 given on E13.5 in mice is sufficient to cause an expanded NPC population in 2-month old offspring, indicating that dysregulated NPC behavior may endure long after *in utero* exposure to inflammation (53). The ramifications of dysregulated NPC behavior and altered corticogenesis are not fully established, but one possible repercussion is exhaustion of NPC pools in older adult offspring (54). Another possible consequence is that cortical structural defects resulting from aberrant NPC behavior may be an etiological factor linking MIA with poor neurobehavioral outcomes in offspring. In support of this possibility, increased brain volume, head circumference, and number of cortical neurons are found in a subset of patients diagnosed with ASD (55, 56).

The evolutionarily conserved Notch signaling pathway is a major contributor to cell fate determination and patterning in the developing nervous system, including neurogenesis in embryonic brains, maintenance of NPC populations, and specification of glia (27). Mice lacking *Notch1, Dll1*, or *Rbpj* (which encodes a key nuclear component of the canonical Notch pathway) exhibit early embryonic lethality with compromised nervous system development, including rapid depletion of NPCs and precocious neuronal differentiation (57, 58), suggesting that signaling through Notch1 and Dll1 is critical for proliferation and maintenance of NPCs. The role of Notch3 in NPC biology is less clear. Notch3 is expressed in NPCs, but mice lacking Notch3 are viable and their brain development appears normal (59). In some studies, Notch3 is found to promote NPC proliferation similar to Notch1; other studies have reported Notch3 antagonizes Notch1, thereby inhibiting NPC proliferation and stimulating differentiation of NPCs (60). Intriguingly, we observed increased expression of *Notch1* and *Dll1*, and decreased expression of *Notch3* in conceptuses following MIA. Consistent with our findings, elevated expression of several Notch signaling components (e.g., *Notch1, Dll1*) is evident in inflammatory conditions such as rheumatoid arthritis and systemic lupus erythematosus, and *de novo* production of Notch ligands and receptors is increased following activation of TLR pathways [reviewed in (61)]. Moreover, direct infection of human NPCs with Zika virus is sufficient to induce Notch signaling; proper differentiation and improved viability of Zika-infected NPCs is restored using a Notch pathway inhibitor, DAPT (62). We therefore propose that dysregulated expression of Notch signaling components following MIA may contribute, at least in part, to the sustained increase in NPC proliferation and increased thickness of cortical layers during fetal development.

In conclusion, we have uncovered a comprehensive profile of gene expression changes in the rat conceptus following an early gestational exposure to PolyI:C and correlated these changes to sustained dysregulation of cortical structure and NPC function. PolyI:C and other immunogens (e.g., lipopolysaccharide) elicit strong, short-lived innate immune responses, and have an advantage of controlling the onset and severity of MIA within defined periods of embryogenesis. A limitation of these models is that they may not fully recapitulate the broad and potentially long-lasting immune response characteristic of live pathogens. Despite this limitation, many distinct types of bacterial and viral infections predispose to neuropsychiatric deficiencies in humans at various stages in pregnancy, and behavioral phenotypes are comparable in rodents challenged prenatally with PolyI:C, lipopolysaccharide, and active pathogens (e.g., influenza A) (63). This suggests that maternal immune responses provoked by distinct pathogens and immunogens may converge to impact development of the primordial nervous system, with implications for long-term cognitive function and behavioral phenotype. In future studies, it will be enticing to dissect the impact that MIA exerts on pathways vital for nascent nervous system development, such as dysregulated expression of Notch signaling components, which may underlie the pathogenesis of neurodevelopmental disorders caused by distinct stimuli. For example, in another rodent model that elicits behaviors reminiscent of ASD, inhibition of excessive Notch signaling alleviates ASD-like behaviors in offspring (64). Therefore, this pathway-specific approach may yield new therapeutic strategies that selectively target the molecular underpinnings of abnormalities in cortical development and offspring behavior following prenatal exposure to immunogens.

## Data Availability Statement

The datasets generated for this study can be found in the Gene Expression Omnibus (GSE145167).

## Ethics Statement

The animal study was reviewed and approved by the University of Western Ontario Animal Care Committee under guidelines from the Canadian Council of Animal Care.

## Author Contributions

KB, DH, FH, SS, and SR contributed to the overall approach and design of experiments. KB and DH conducted and analyzed the experiments. NR was involved in drafting and critically revising the manuscript for intellectual content. KB, DH, and SR wrote the manuscript.

## Conflict of Interest

The authors declare that the research was conducted in the absence of any commercial or financial relationships that could be construed as a potential conflict of interest.

## References

[1] Gumusoglu SB Stevens HE. Maternal inflammation and neurodevelopmental programming: a review of preclinical outcomes and implications for translational psychiatry. Biol Psychiatry. (2018) 85:107–21. 10.1016/j.biopsych.2018.08.008 30318336

[2] Brown AS Vinogradov S Kremen WS Poole JH Deicken RF Penner JD. Prenatal exposure to maternal infection and executive dysfunction in adult schizophrenia. Am J Psychiatry. (2009) 166:683–90. 10.1176/appi.ajp.2008.08010089 19369317 PMC2885160

[3] Parker-Athill EC Tan J. Maternal immune activation and autism spectrum disorder: interleukin-6 signaling as a key mechanistic pathway. NeuroSignals. (2011) 18:113–28. 10.1159/000319828 20924155 PMC3068755

[4] Sun Y Vestergaard M Christensen J Nahmias AJ Olsen J. Prenatal exposure to maternal infections and epilepsy in childhood: a population-based cohort study. Pediatrics. (2008) 121:e110–7. 10.1542/peds.2007-2316 18450853

[5] Carvey PM Chang Q Lipton JW Ling Z. Prenatal exposure to the bacteriotoxin lipopolysaccharide leads to long-term losses of dopamine neurons in offspring: a potential, new model of Parkinson's disease. Front Biosci. (2003) 8:826–37. 10.2741/1158 12957870

[6] Hoeijmakers L Heinen Y van Dam AM Lucassen PJ Korosi A. Microglial priming and alzheimer's disease: a possible role for (early) immune challenges and epigenetics? Front Hum Neurosci. (2016) 10:15. 10.3389/fnhum.2016.00398 27555812 PMC4977314

[7] Boksa P. Effects of prenatal infection on brain development and behavior: a review of findings from animal models. Brain Behav Immun. (2010) 24:881–97. 10.1016/j.bbi.2010.03.005 20230889

[8] Meyer U Feldon J. To poly(I:C) or not to poly(I:C): advancing preclinical schizophrenia research through the use of prenatal immune activation models. Neuropharmacology. (2012) 62:1308–21. 10.1016/j.neuropharm.2011.01.009 21238465

[9] Field R Campion S Warren C Murray C Cunningham C. Systemic challenge with the TLR3 agonist polyI:C induces amplified IFNα/β and IL-1β responses in the diseased brain and exacerbates chronic neurodegeneration. Brain Behav Immun. (2010) 24:996–1007. 10.1016/j.bbi.2010.04.004 20399848 PMC3334265

[10] Smith SEP Li J Garbett K Mirnics K Patterson PH. Maternal immune activation alters fetal brain development through interleukin-6. J Neurosci. (2007) 27:10695–702. 10.1523/JNEUROSCI.2178-07.2007 17913903 PMC2387067

[11] Meyer U Nyffeler M Yee BK Knuesel I Feldon J. Adult brain and behavioral pathological markers of prenatal immune challenge during early/middle and late fetal development in mice. Brain Behav Immun. (2008) 22:469–86. 10.1016/j.bbi.2007.09.012 18023140

[12] Careaga M Murai T Bauman MD. Maternal immune activation and autism spectrum disorder: from rodents to nonhuman and human primates. Biol Psychiatry. (2017) 81:391–401. 10.1016/j.biopsych.2016.10.020 28137374 PMC5513502

[13] Goldman-Rakic PS. Cellular basis of working memory. Neuron. (1995) 14:477–85. 10.1016/0896-6273(95)90304-6 7695894

[14] Martynoga B Drechsel D Guillemot F. Molecular control of neurogenesis: a view from the mammalian cerebral cortex. Cold Spring Harb Perspect Biol. (2012) 4:a008359. 10.1101/cshperspect.a008359 23028117 PMC3475166

[15] Rice D Barone S. Critical periods of vulnerability for the developing nervous system: evidence from humans and animal models. Environ Health Perspect. (2000) 108:511–33. 10.1289/ehp.00108s3511 10852851 PMC1637807

[16] Baines KJ Rampersaud AM Hillier DM Jeyarajah MJ Grafham GK Lacefield JC. Antiviral inflammation during early pregnancy reduces placental and fetal growth trajectories. J Immunol. (2020) 204:694–706. 10.4049/jimmunol.1900888 31882516

[17] Bloise E Petropoulos S Iqbal M Kostaki A Ortiga-Carvalho TM Gibb W. Acute effects of viral exposure on p-glycoprotein function in the mouse fetal blood-brain barrier. Cell Physiol Biochem. (2017) 41:1044–50. 10.1159/000461569 28222448

[18] Forrest CM Khalil OS Pisar M Smith RA Darlington LG Stone TW. Prenatal activation of Toll-like receptors-3 by administration of the viral mimetic poly(I:C) changes synaptic proteins, N-methyl-D-aspartate receptors and neurogenesis markers in offspring. Mol Brain. (2012) 5:22. 10.1186/1756-6606-5-22 22681877 PMC3496691

[19] Huang DW Sherman BT Lempicki RA. Systematic and integrative analysis of large gene lists using DAVID bioinformatics resources. Nat Protoc. (2009) 4:44–57. 10.1038/nprot.2008.211 19131956

[20] Campos LS. Neurospheres: insights into neural stem cell biology. J Neurosci Res. (2004) 78:761–9. 10.1002/jnr.20333 15505793

[21] Ghosh A Greenberg ME. Distinct roles for bFGF and NT-3 in the regulation of cortical neurogenesis. Neuron. (1995) 15:89–103. 7619533 10.1016/0896-6273(95)90067-5

[22] Schneider CA Rasband WS Eliceiri KW. NIH Image to ImageJ: 25 years of image analysis. Nature methods (2012) 9:671–5. 22930834 10.1038/nmeth.2089 PMC5554542

[23] Rose DR Careaga M van de Water J McAllister K Bauman MD Ashwood P. Long-term altered immune responses following fetal priming in a non-human primate model of maternal immune activation. Brain Behav Immun. (2017) 63:60–70. 10.1016/j.bbi.2016.11.020 27876552 PMC5432383

[24] Richetto J Chesters R Cattaneo A Labouesse MA Gutierrez AMC Wood TC. Genome-wide transcriptional profiling and structural magnetic resonance imaging in the maternal immune activation model of neurodevelopmental disorders. Cereb Cortex. (2017) 27:3397–413. 10.1093/cercor/bhw320 27797829

[25] Weber-Stadlbauer U Richetto J Labouesse MA Bohacek J Mansuy IM Meyer U. Transgenerational transmission and modification of pathological traits induced by prenatal immune activation. Mol Psychiatry. (2017) 22:102–12. 10.1038/mp.2016.41 27021823

[26] Workman AD Charvet CJ Clancy B Darlington RB Finlay BL. Modeling transformations of neurodevelopmental sequences across mammalian species. J Neurosci. (2013) 33:7368–83. 10.1523/JNEUROSCI.5746-12.2013 23616543 PMC3928428

[27] Pierfelice T Alberi L Gaiano N. Notch in the vertebrate nervous system: an old dog with new tricks. Neuron. (2011) 69:840–55. 10.1016/j.neuron.2011.02.031 21382546

[28] Meyer U. Prenatal poly(I:C) exposure and other developmental immune activation models in rodent systems. Biol Psychiatry. (2014) 75:307–15. 10.1016/j.biopsych.2013.07.011 23938317

[29] Mian MF Ahmed AN Rad M Babaian A Bowdish D Ashkar AA. Length of dsRNA (poly I:C) drives distinct innate immune responses, depending on the cell type. J Leukoc Biol. (2013) 94:1025–36. 10.1189/jlb.0312125 23911868

[30] Malkova NV Yu CZ Hsiao EY Moore MJ Patterson PH. Maternal immune activation yields offspring displaying mouse versions of the three core symptoms of autism. Brain Behav Immun. (2012) 26:607–16. 10.1016/j.bbi.2012.01.011 22310922 PMC3322300

[31] Murray C Griffin ÉW O'Loughlin E Lyons A Sherwin E Ahmed S. Interdependent and independent roles of type I interferons and IL-6 in innate immune, neuroinflammatory and sickness behaviour responses to systemic polyI:C. Brain Behav Immun. (2015) 48:274–86. 10.1016/j.bbi.2015.04.009 25900439 PMC4521083

[32] Choi J Hild S Zeiher J Schaub P Rubio-abadal A Yefsah T. The maternal interleukin-17a pathwayin mice promotes autism-like phenotypes in offspring. Science. (2016) 354:933–9. 10.1126/science.aad0314 26822608 PMC4782964

[33] Hsiao EY Patterson PH. Activation of the maternal immune system induces endocrine changes in the placenta via IL-6. Brain Behav Immun. (2011) 25:604–15. 10.1016/j.bbi.2010.12.017 21195166 PMC3081363

[34] Garbett KA Hsiao EY Kálmán S Patterson PH Mirnics K. Effects of maternal immune activation on gene expression patterns in the fetal brain. Transl Psychiatry. (2012) 2:e98. 10.1038/tp.2012.24 22832908 PMC3337077

[35] Kim H Chen L Lim G Sung B Wang S Mccabe MF. Brain indolamine 2,3-dioxygenase contributes to the comorbidity of pain and depression. J Clin Invest. (2012) 122:2940–54. 10.1172/JCI61884DS1 22751107 PMC3408737

[36] McColl SR Mahalingam S Staykova M Tylaska LA Fisher KE Strick CA. Expression of rat I-TAC/CXCL11/SCYA11 during central nervous system inflammation: Comparison with other CXCR3 ligands. Lab Investig. (2004) 84:1418–29. 10.1038/labinvest.3700155 15322564

[37] Buchrieser J Degrelle SA Couderc T Nevers Q Disson O Manet C. IFITM proteins inhibit placental syncytiotrophoblast formation and promote fetal demise. Science. (2019) 365:176–80. 10.1126/science.aaw7733 31296770

[38] Bonow RH Aïd S Zhang Y Becker KG Bosetti F. The brain expression of genes involved in inflammatory response, the ribosome, and learning and memory is altered by centrally injected lipopolysaccharide in mice. Pharmacogenomics J. (2009) 9:116–26. 10.1038/tpj.2008.15 18957951 PMC2728029

[39] Silverman HA Dancho M Regnier-Golanov A Nasim M Ochani M Olofsson PS. Brain region-specific alterations in the gene expression of cytokines, immune cell markers and cholinergic system components during peripheral endotoxin-induced inflammation. Mol Med. (2014) 20:601–11. 10.2119/molmed.2014.00147 25299421 PMC4365063

[40] Sousa C Golebiewska A Poovathingal SK Kaoma T Pires-afonso Y Martina S. Single-cell transcriptomics reveals distinct inflammation-induced microglia signatures. EMBO Rep. (2018) 19:1–7. 10.15252/embr.201846171 30206190 PMC6216255

[41] Oskvig DB Elkahloun AG Johnson KR Phillips TM Herkenham M. Maternal immune activation by LPS selectively alters specific gene expression profiles of interneuron migration and oxidative stress in the fetus without triggering a fetal immune response. Brain Behav Immun. (2012) 26:623–34. 10.1016/j.bbi.2012.01.015 22310921 PMC3285385

[42] Lombardo MV Moon HM Su J Palmer TD Courchesne E Pramparo T. Maternal immune activation dysregulation of the fetal brain transcriptome and relevance to the pathophysiology of autism spectrum disorder. Mol Psychiatry. (2018) 23:1001–13. 10.1038/mp.2017.15 28322282 PMC5608645

[43] Pang ZP Bacaj T Yang X Zhou P Xu W Südhof TC. Doc2 supports spontaneous synaptic transmission by a Ca2+-independent mechanism. Neuron. (2011) 70:244–51. 10.1016/j.neuron.2011.03.011 21521611 PMC3102832

[44] Masuda T Taniguchi M. Contribution of semaphorins to the formation of the peripheral nervous system in higher vertebrates. Cell Adhes Migr. (2016) 10:593–603. 10.1080/19336918.2016.1243644 27715392 PMC5160040

[45] Parekh PK Becker-Krail D Sundaravelu P Ishigaki S Okado H Sobue G. Altered GluA1 (Gria1) function and accumbal synaptic plasticity in the ClockΔ19 model of bipolar Mania. Biol Psychiatry. (2018) 84:817–26. 10.1016/j.biopsych.2017.06.022 28780133 PMC5745309

[46] Rannals MDD Hamersky GRR Page SCC Campbell MNN Briley A Gallo RAA. Psychiatric risk gene transcription factor 4 regulates intrinsic excitability of prefrontal neurons via repression of SCN10a and KCNQ1. Neuron. (2016) 90:43–55. 10.1016/j.neuron.2016.02.021 26971948 PMC4824652

[47] Tsou JH Yang YC Pao PC Lin HC Huang NK Lin ST. Important roles of ring finger protein 112 in embryonic vascular development and brain functions. Mol Neurobiol. (2017) 54:2286–300. 10.1007/s12035-016-9812-7 26951452

[48] Ben-Reuven L Reiner O. Dynamics of cortical progenitors and production of subcerebral neurons are altered in embryos of a maternal inflammation model for autism. Mol Psychiatry. (2019). 10.1038/s41380-019-0594-y. [Epub ahead of print]. 31740755

[49] Smith SEP Elliott RM Anderson MP. Maternal immune activation increases neonatal mouse cortex thickness and cell density. J Neuroimmune Pharmacol. (2012) 7:529–32. 10.1007/s11481-012-9372-1 22570011 PMC3672058

[50] Le Belle JE Sperry J Ngo A Ghochani Y Laks DR López-Aranda M. Maternal inflammation contributes to brain overgrowth and autism-associated behaviors through altered redox signaling in stem and progenitor cells. Stem Cell Rep. (2014) 3:725–34. 10.1016/j.stemcr.2014.09.004 25418720 PMC4235743

[51] Chavez-Valdez R Mottahedin A Stridh L Yellowhair TR Jantzie LL Northington FJ. Evidence for sexual dimorphism in the response to TLR3 activation in the developing neonatal mouse brain: a pilot study. Front Physiol. (2019) 10:306. 10.3389/fphys.2019.00306 30971945 PMC6443881

[52] Loram LC Sholar PW Taylor FR Wieseler J Babb JA Strand A. Sex and estradiol influence glial pro-inflammatory responses to lipopolysaccharide in rats. Psychoneuroendocrinology. (2012) 37:1688–99. 10.1016/j.psyneuen.2012.02.018.Sex 22497984 PMC3417083

[53] Gallagher D Norman AA Woodard CL Yang G Fujitani M Vessey JP. Transient maternal IL-6 mediates long-lasting changes in neural stem cell pools by deregulating an endogenous self-renewal pathway. Cell Stem Cell. (2013) 13:564–76. 10.1016/j.stem.2013.10.002 24209760

[54] Development.Storer MA Gallagher D Fatt MP Simonetta JV Kaplan DR Miller FD. Interleukin-6 regulates adult neural stem cell numbers during normaland abnormal post-natal development. Stem Cell Rep. (2018) 10:1464–80. 10.1016/j.stemcr.2018.03.008 29628394 PMC5995693

[55] Courchesne E Mouton PR Calhoun ME Semendeferi K Hallet MJ Barnes CC. Neuron number and size in prefrontal cortex of children with autism. J Am Med Assoc. (2011) 306:2001–10. 10.1001/jama.2011.1638 22068992

[56] Pramparo T Lombardo MV Campbell K Barnes CC Marinero S Solso S. Cell cycle networks link gene expression dysregulation, mutation and brain maldevelopment in autistic toddlers. Mol Syst Biol. (2015) 11:841. 10.15252/msb.20156108 26668231 PMC4704485

[57] Imayoshi I Sakamoto M Yamaguchi M Mori K Kageyama R. Essential roles of Notch signaling in maintenance of neural stem cells in developing and adult brains. J Neurosci. (2010) 30:3489–98. 10.1523/JNEUROSCI.4987-09.2010 20203209 PMC6634119

[58] Hitoshi S Alexson T Tropepe V Donoviel D Elia AJ Nye JS. Notch pathway molecules are essential for the maintenance, but not the generation, of mammalian neural stem cells. Genes Dev. (2002) 16:846–58. 10.1101/gad.975202 11937492 PMC186324

[59] Krebs LT Xue Y Norton CR Sundberg JP Beatus P Lendahl U. Characterization of notch3 -deficient mice : normal embryonic development and absence of genetic interactions with a notch1 mutation. Genesis. (2003) 37:139–43. 10.1002/gene.10241 14595837

[60] Rusanescu G Mao J. Notch3 is necessary for neuronal differentiation and maturation in the adult spinal cord. J Cell Mol Med. (2014) 18:2103–16. 10.1111/jcmm.12362 25164209 PMC4244024

[61] Shang Y Smith S Hu X. Role of Notch signaling in regulating innate immunity and inflammation in health and disease. Protein Cell. (2016) 7:159–74. 10.1007/s13238-016-0250-0 26936847 PMC4791423

[62] Ferraris P Cochet M Hamel R Gladwyn-Ng I Alfano C Diop F. Zika virus differentially infects human neural progenitor cells according to their state of differentiation and dysregulates neurogenesis through the Notch pathway. Emerg Microbes Infect. (2019) 8:1003–16. 10.1080/22221751.2019.1637283 31282298 PMC6691766

[63] Arsenault D St-Amour I Cisbani G Rousseau LS Cicchetti F. The different effects of LPS and polyI:C prenatal immune challenges on the behavior, development and inflammatory responses in pregnant mice and their offspring. Brain Behav Immun. (2014) 38:77–90. 10.1016/j.bbi.2013.12.016 24384468

[64] Zhang Y Xiang Z Jia Y He X Wang L Cui W. The Notch signaling pathway inhibitor Dapt alleviates autism-like behavior, autophagy and dendritic spine density abnormalities in a valproic acid-induced animal model of autism. Prog Neuropsychopharmacol Biol Psychiatry. (2019) 94:109644. 10.1016/j.pnpbp.2019.109644 31075347

