# Supplementary figures and images for: Maternal Immune Activation Alters Fetal Brain Development and Enhances Proliferation of Neural Precursor Cells in Rats
Source: Front Immunol. 2020 Jun 9;11:1145. doi: 10.3389/fimmu.2020.01145 (PMC7295982; doi:10.3389/fimmu.2020.01145)

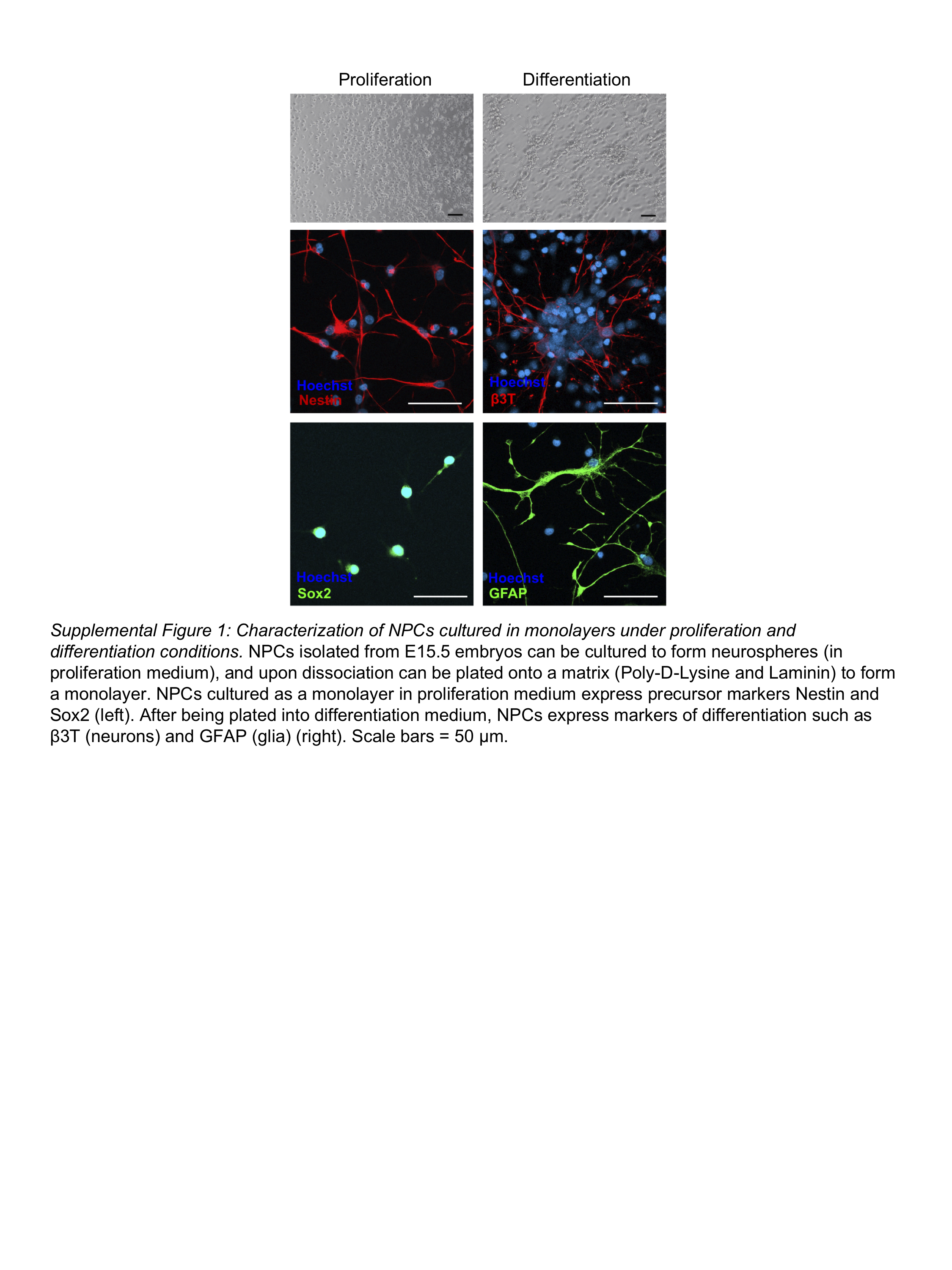

Supplement: Supplementary file 1 [file Image_1.TIFF]
